# Repurposing the prostaglandin analogue treprostinil and the calcium-sensing receptor modulator cinacalcet to revive cord blood as an alternate source of hematopoietic stem and progenitor cells for transplantation

**DOI:** 10.3389/fphar.2024.1444311

**Published:** 2025-01-09

**Authors:** Michaela Prchal-Murphy, Julia Zehenter, Marlene Fischer, Anita Pirabe, Madeleine Themanns, Behnaz Afrashteh, Eva Maria Putz, Karoline Kollmann, José Basílio, Manuel Salzmann, Wolfgang Strohmaier, Günther Krumpl, Alex Farr, Veronika Sexl, Michael Freissmuth, Eva Zebedin-Brandl

**Affiliations:** ^1^ Department for Biological Sciences and Pathobiology, Pharmacology and Toxicology, University of Veterinary Medicine, Vienna, Austria; ^2^ Institute of Pharmacology and the Gaston H. Glock Research Laboratories for Exploratory Drug Development, Centre of Physiology and Pharmacology, Medical University of Vienna, Vienna, Austria; ^3^ Department for Vascular Biology and Thrombosis Research, Medical University of Vienna, Vienna, Austria; ^4^ Institute of Pathophysiology and Allergy Research, Center for Pathophysiology, Infectiology and Immunology, Medical University of Vienna, Vienna, Austria; ^5^ Department of Vascular Biology and Thrombosis Research, Center for Physiology and Pharmacology, Medical University Vienna, Vienna, Austria; ^6^ Division of Cardiology, Department of Internal Medicine II, Medical University of Vienna, Vienna, Austria; ^7^ AOP Orphan Pharmaceuticals GmbH, Vienna, Austria; ^8^ AOP Health International Management AG, Ruggell, Liechtenstein; ^9^ Department of Obstetrics and Gynecology, Division of Obstetrics and Feto-Maternal Medicine, Medical University of Vienna, Vienna, Austria

**Keywords:** CD34^+^ HSPCs, cord blood, stem cell transplantation, drug repurposing, engraftment efficiency, bone marrow reconstitution, differentiation

## Abstract

**Objective:**

The expanding field of hematopoietic cell transplantation (HCT) for non-malignant diseases, including those amenable to gene therapy or gene editing, faces challenges due to limited donor availability and the toxicity associated with cell collection methods. Umbilical cord blood (CB) represents a readily accessible source of hematopoietic stem and progenitor cells (HSPCs); however, the cell dose obtainable from a single cord blood unit is frequently insufficient. This limitation can be addressed by enhancing the potency of HSPCs, specifically their capacity to reconstitute hematopoiesis. In our study, we investigated the combined effects of treprostinil, a prostaglandin analog, and cinacalcet, a calcium-sensing receptor modulator, on the reconstitution of hematopoiesis.

**Methods:**

A Lineage Cell Depletion Kit was employed to isolate lineage-negative (lin^−^) HSPCs from mouse bone marrow. A Human CB CD34 Positive Selection Kit was utilized to isolate CD34^+^ cells from the CB of healthy donors. *In vitro*, the effects of treprostinil, cinacalcet, and their combination on the migration, adhesion, and differentiation of HSPCs were assessed. *In vivo*, homing and engraftment were examined. Eight-week-old female and male C57BL/6J, BALB/c, or female NSG mice served as recipient models.

**Results:**

When administered concomitantly, treprostinil and cinacalcet exhibited mutual antagonism: the survival of recipient animals was lower when both drugs were administered together compared to either agent alone. Conversely, a sequential regimen involving priming with treprostinil/forskolin followed by cinacalcet treatment *in vivo* enhanced survival, irrespective of whether hematopoiesis was reconstituted by human or murine HSPCs. *In vitro* assays demonstrated enhanced migration and adhesion in response to the presence of treprostinil and cinacalcet, suggesting potential synergistic effects. Colony formation confirmed synergism.

**Conclusion:**

Augmenting the bone marrow reconstitution potential of HSPCs with treprostinil and cinacalcet shows promise for rescuing patients undergoing HCT. This approach is particularly beneficial for those patients at high risk of transplant failure due to limited numbers of available HSPCs. Furthermore, enhancing the potency of HSPCs has the potential to alleviate the burden and risks associated with HSPC donation, as it would reduce the number of cells needed for collection.

## 1 Introduction

Drug repurposing, or repositioning, is a strategy where existing drugs are used for new therapeutic purposes beyond their original indications. This approach leverages known safety profiles, cutting time and costs in drug development, and has enabled treatments for various conditions, including rare diseases and cancers (Pushpakom et al., 2019; Pillaiyar et al., 2020).

Transplantation of hematopoietic stem and progenitor cells (HSPCs) has been continuously improved over the past 6 decades and is currently the standard of care for treating of various malignant diseases, inherited immune system or metabolic disorders and autoimmune diseases (Juric et al., 2016). These life-threatening or debilitating diseases may be treated or alleviated by hematopoietic cell transplants (HCTs), which involve harvesting CD34^+^ HSPCs from bone marrow (BM), mobilized peripheral blood (PB), or umbilical cord blood (CB) at birth.

The source of CD34^+^ cells influences their cellular characteristics as well as their clinical applications (Panch et al., 2017). PB is the most commonly utilized source, followed by BM (Sun et al., 2022), and both approaches can impose significant demands on the donor. Harvesting HSPCs from bone marrow requires general anaesthesia and carries risks like infection, bleeding, and pain. In contrast, collecting HSPCs from peripheral blood (PB) involves administering a mobilizing agent, such as granulocyte-colony stimulating factor (G-CSF), which may cause flu-like symptoms, bone pain, and insomnia. A major risk of both PB and BM transplants is graft-versus-host disease (GvHD), caused by T cells in the transplant.

CB offers benefits including rapid availability of donors, less strict HLA-matching demands, and low rates of graft-versus-host disease GVHD as compared to BM and mobilized PB (Panch et al., 2017; Sun et al., 2022). However, a significant concern among clinicians regarding the use of CB hematopoietic cell transplantation (CB-HCT) is the limited number of HSPCs present in CB donations. This may result in delayed neutrophil recovery, increased risk of transplant-related infection and mortality, and proneness to graft failure. Therefore, a single CB unit is often insufficient for transplantation, necessitating the use of two CB units. Consequently, the quality and utility of CB as a source of HSPCs are diminished. Efforts to improve CB engraftments include the *ex vivo* expansion of HSPCs and the enhancement of their homing capabilities to the BM of the recipient (Sun et al., 2022).

Upon enhancing the “potency” of available HSPCs, G protein-coupled receptors (GPCRs) rise as a compelling target of interest due to their easily accessible seven-transmembrane protein structure and their variety of downstream signaling pathways (Kamato et al., 2015).

Activation of five GPCRs has been linked to enhanced engraftment of HSPCs. The G_s_-coupled E prostanoid receptors 2 and 4, EP2R and EP4R (Hoggatt et al., 2009; Ikushima et al., 2013), the G_i_/G_q_-coupled calcium-sensing receptor (CaSR) (Adams et al., 2006), the G_i_-coupled receptor for stromal cell-derived factor-1 (SDF1 = CXCL12) (Broxmeyer et al., 2005) CXCR4, and C3AR1, the G_i_/G_12/13_-coupled receptor for complement factor C3a (Brunstein et al., 2013). Two licensed drugs, the stable prostacyclin (IP) analog treprostinil (Whittle et al., 2012) and the CaSR-sensitizer cinacalcet (Messa et al., 2008; Chavez-Abiega et al., 2020), can easily target two of these receptors.

Treprostinil, an EP2R, EP4R, and IPR agonist (Whittle et al., 2012) has been used to treat pulmonary hypertension for over 2 decades, with well-understood pharmacodynamics and pharmacokinetics (Skoro-Sajer et al., 2008). It increases CXCR4 expression, enhancing CXCR4-mediated migration, adhesion, and engraftment. Treprostinil also boosts cAMP in HSPCs, an effect amplified by co-administration with adenylate cyclase sensitizers like forskolin, which makes AC isoforms more responsive to Gαs, raising cAMP levels and promoting differentiation without affecting the cell cycle or apoptosis (Kazemi et al., 2016; Zebedin-Brandl et al., 2020).

The activity of the CaSR can be modulated by the allosteric activator cinacalcet, which was approved for the treatment of hyperparathyroidism in 2004. Secondary hyperparathyroidism is a frequent complication of chronic kidney disease (Messa et al., 2008). By increasing the sensitivity of CaSR to extracellular calcium, cinacalcet lowers PTH secretion from the parathyroid glands. This action effectively reduces serum calcium levels in addition to phosphorus levels (Lam et al., 2011; Pereira et al., 2018; Eidman and Wetmore, 2018; Akizawa et al., 2020). Over the past 2 decades, several million patients have been treated with the drug, providing valuable insights into its pharmacological effects in humans.

Cinacalcet acts in a cell-autonomous manner on HSPCs. Considering that calcium levels mold the bone endosteal surface into a niche for circulating HSPCs, the concentration of calcium ions at the endosteum is critical for effective homing and lodgment of HSPCs (Silver et al., 1988). Therefore, the presence of cinacalcet primes HSPCs for homing into the BM of recipient animals directly (Lam et al., 2011). Similar to the action of treprostinil (Kazemi et al., 2016), the effect of cinacalcet on HSPC engraftment is also in part dependent on CXCR4 (Lam et al., 2011).

Drug combinations are appealing as they can leverage synergy, reduce doses, and minimize side effects, making them common in treatments for conditions like hypertension, heart failure, and cancer (Jia et al., 2009).

Here, we analyzed the combination of cinacalcet and treprostinil, comparing their individual and combined effects on HSPC migration, adhesion, and differentiation *in vitro*, and on homing and BM reconstitution *in vivo*. The optimal regimen was found when HSPCs primed with treprostinil/forskolin were transplanted into recipients subsequently treated with cinacalcet. This sequential approach supported survival in lethally irradiated mice at low cell doses. However, simultaneous administration led to antagonism and poor survival, as predicted by *in vitro* results.

## 2 Methods

### 2.1 Isolation of murine and human HSPCs

Murine BM and human CB HSPCs were harvested as previously reported (Kazemi et al., 2016; Zebedin-Brandl et al., 2020): briefly, bone marrow cells were flushed from the femora and tibiae of donor mice. After osmotically induced lysis of erythrocytes, lineage-negative (lin^–^) and lineage-positive (lin^+^) cells were separated with antibody-coated magnetic beads (Lineage Cell Depletion Kit, Miltenyi Biotec GmbH, Bergisch Gladbach, Germany). Human HSPCs were isolated from donated umbilical cord blood: mononuclear cells were enriched using Lymphoprep (StemCell Technologies, Vancouver, Canada), contaminating erythrocytes were removed by osmotic cell lysis and CD34^+^ cells were retrieved by sorting with antibody-coated magnetic beads (CD34 MicroBead Kit Ultra Pure, Miltenyi Biotec GmbH). After collection, cells were stored in freezing medium (90% FBS, 10% DMSO) in liquid nitrogen.

### 2.2 Culture and priming of murine and human HSPCs

Murine HSPCs were cultured in StemSpan SFEM medium containing benzylpenicillin and streptomycin (0.5 mg L^−1^ each), murine stem cell factor, human FLT3, interleukin (IL)-11 (50 μg L^−1^ each), and murine IL-3 (150 μg L^−1^) (PeproTech, Vienna, Austria) (Kazemi et al., 2016; Zebedin-Brandl et al., 2020). Human HSPCs were cultured in IMDM medium containing benzylpenicillin and streptomycin (0.5 mg L^−1^ each), human FLT3, thrombopoietin, and stem cell factor (50 ng mL^−1^ each; R&D Systems, Inc., Minneapolis, MN). For *in vitro* priming, murine and human cells were incubated for 1 h at 37°C in their respective culture medium in the absence (untreated controls) or in the presence of 2.5 µM cinacalcet (Lam et al., 2011), or 10 µM treprostinil and 30 µM forskolin (Kazemi et al., 2016), or the combination of 2.5 µM cinacalcet, 10 µM treprostinil and 30 µM forskolin.

### 2.3 Analysis of gene expression by quantitative PCR

Total mRNA from human HSPCs was isolated using the RNeasy Micro Kit (QIAGEN, Hilden, Germany) and reverse transcribed into complementary DNA (cDNA) using RevertAid First Strand cDNA Synthesis Kit (Thermo Scientific, Vienna, Austria). Quantitative PCR (qPCR) was performed using Maxima SYBR Green/ROX qPCR Master Mix (ThermoFisher Scientific, Vienna, Austria) in 41 cycles (15-s denaturation at 95°C, annealing at 60°C for 30 s, extension 72°C for 30 s) to assess the levels of human transcripts encoding adenylyl cyclases 1 to 9 (AC1 to AC9). Human hypoxanthine phosphoribosyltransferase (HPRT) was used as a reference gene for qPCR. Primer efficiency was verified by serial dilution of the template. Each reaction was performed in triplicates. Relative abundance of transcripts was calculated using the 2^−ΔΔCT^ method (gene-specific expression level relative to that of the reference gene). The primers (sequences listed in Table 1) were purchased from Microsynth AG (Balgach, Switzerland).

**TABLE 1:** Primer sequences. Listed are all forward and reverse primer sequences used.

Primers that were used for amplification by qPCR of AC subtypes in human HSPC RNA samples	
Target and houskeeping genes	Forward & reverse
AC1	5′-ATG AGC TCT TCG GCA AGT TC-3′
	5′-CCG ACA CGC AGT AGC AG-3′
AC2	5′-CTT GTT GCC ATG GGA TAC CT-3′
	5′-CCA CGA AGA TGA AGA GC-3′
AC3	5′-AGG AGG AGC TCA AGG GGA T-3′
	5′-TTG CGG TCA GCC ATG TAG TA-3′
AC4	5′-ACT GCT GAT GAC CCG TTA CC-3′
	5′-CTG AAG GAA GCC AGG GTC T-3′
AC5	5′-AGG ACC AGT TCC TGC TGA AG-3′
	5′-TCC TGG AAA GCC TGT CTC TG-3′
AC6	5′-CTT GCA TTT GAT CTT GGC CT-3′
	5′-GGA TAG TGT GTG GAG ATG CC-3′
AC7	5′-ACT GTT CTC CCA AGG AGC TG-3′
	5′-CAC AGT AGT AGC AGT CGC CG-3′
AC8	5′-TCC TCC AAG TGG TCA TAC CC-3′
	5′-TGA AGA TTC CAG CTG TGT TCA-3′
AC9	5′-GAC CGG TAC GAA ATG GAA GA-3′
	5′-CTT GGC TCT CTG ACC CGA TA-3′
HRPT	5′-CAC CCT TTC CAA ATC CTC AG-3′
	5′-CTC CGT TAT GGC GAC CC-3′

### 2.4 [^3^H] cAMP accumulation

Accumulation of cAMP was determined as previously reported (Kazemi et al., 2016). Briefly, human HSPCs were incubated with medium containing [^3^H] adenine (1 μCi mL^−1^) (PerkinElmer, Boston, MA) for 16 h and subsequently resuspended in medium containing phosphodiesterase inhibitors Ro 20-1724 and isobutylmethylxanthine (Calbiochem/EMD Millipore, Darmstadt, Germany) at 100 μM and 125 μM, respectively. In parallel, cyclic AMP levels were assessed in cells, that had been pre-incubated for 24 h with pertussis toxin (PTX, 100 ng mL^−1^) (Sigma-Aldrich, St. Louis, MO). cAMP formation was stimulated by the addition of 10 µM treprostinil and 30 µM forskolin, 2.5 µM cinacalcet or the combination thereof. After an incubation for 30 min at 37°C, the cells were lysed in ice-cold 2.5% perchloric acid containing 0.1 mM cAMP for 30 min at 4°C; the solution was neutralized with 4.2 M KOH. ATP and cAMP were separated by sequential chromatography on columns containing Dowex 50-X4 (Sigma-Aldrich) and neutral alumina (Kazemi et al., 2016). The accumulated [^3^H] cAMP was quantified by liquid scintillation counting.

### 2.5 Chemotaxis assay

After *in vitro* priming, human HSPCs (2 × 10^6^ cells) were resuspended in StemSpan SFEM. A cell suspension (0.1 mL containing approximately 1 × 10^5^ cells) was added to the upper chamber of a two-chamber transwell system (Corning Life Sciences, Tewksbury MA). The lower chamber was filled with medium containing 100 ng mL^−1^ SDF-1 (stromal cell-derived factor-1 = CXCL12) or 10 μM CXCR-4 antagonist AMD3100 (=plerixafor; Abcam, Cambridge, United Kingdom) as a chemoattractant. After an incubation for 4 h at 37°C, migrated HSPCs were counted in a Luna automated cell counter (Logos Biosystems, Annandale, VA). Cell count was expressed as percentage of the total number of cells originally added to the upper chamber.

### 2.6 Cell adhesion assay

Cell adhesion was assessed as described in (Lam et al., 2011) with the following modifications: after *in vitro* priming, human HSPCs (5 × 10^2^) were added to 96-well plates (Sarstedt AG & Co. KG, Nümbrecht, Germany) pre-coated with collagen type I (Corning Inc., NY) and incubated for 3 h at 37°C/5% CO_2_ in a humidified atmosphere. Adhesion to 1% bovine serum albumin (Sigma-Aldrich) was quantified to control for nonspecific adhesion. Non-adherent cells were removed with PBS, and adherent cells were counted manually.

### 2.7 Time lapse imaging

Central channels of µ-slide Chemotaxis ibiTreat (ibidi) were coated with 0.41 mg mL^−1^ collagen type I for 1 h at room temperature, washed with PBS and incubated over night at 37°C with 5% CO_2_ together with the respective medium for gas equilibration (Fonseca et al., 2010). On the next day, primed human HSPCs (1.8 × 10^5^) were stained with 0.5 µM CellTracker™ Green CMFDA (Molecular Probes, Vienna, Austria) for 15 min, washed and seeded on the µ-slide. Reservoirs were filled with chemoattractant-free medium and cells were allowed to adhere for 2 h. After adherence of HSPCs, the µ-slide was placed into the ibidi Stage Top Incubation (allowing for an atmosphere of constant gas and humidity), which was mounted on an Olympus X-I71 fluorescence microscope equipped with a ×20 objective (UPlanSApo, NA: 0.75; Olympus, Vienna). A gradient of SDF-1 (100 ng mL^−1^) was generated and cell movement was imaged for 7 h by an fluorescence microscopy system (ibidi). Movement was quantified with the ImageJ plugin “TrackMate” and “Chemotaxis and Migration Tool” provided by ibidi.

### 2.8 Colony formation assay

*In vitro* primed human HSPCs (1 × 10^3^ cells) were resuspended in MethoCult Enriched (StemCell Technologies, Vancouver, Kanada). For sequential treatment, cinacalcet (2.5 µM) was added to the methylcellulose. The cell suspension was plated in 35 mm dishes using a 10 mL syringe and a 21G needle. Cells were incubated at 37°C/5% CO_2_ for 10 days. Individual types of colonies were determined visually according to their morphology.

### 2.9 Mice

Eight-week-old BALB/c, C57BL/6J (CD45.2^+^), B6.SJL-PtrcAPep3B/BoyJ (CD45.1^+^) and NOD.Cg-Prkdcscid Il2rgtm1Wjl/SzJ (NSG) mice were purchased from Charles River Germany (Sulzfeld, Germany) or bred in-house. Housing and husbandry were in accordance with the current recommendations and requirements as defined by the Federation of Laboratory Animal Science Associations (FELASA) in Europe. All recipient mice were randomly allocated to the treatment groups (variation in cell numbers and drug regimen). The allocation was concealed from the person carrying out the tagging. All experiments were performed according to the ARRIVE guideline.

### 2.10 *In vivo* engraftment of murine or human HSPCs

Murine HSPCs and human CB HSPCs were obtained as described above. The transplantation was performed as described in (Zebedin-Brandl et al., 2020): BALB/c or NSG recipient mice were lethally or sub-lethally irradiated (9, 4.5 or 2.4 Gy, split doses, Siemens Primus, 6MV, Siemens Austria). 24 h after irradiation, 1.5 or 2.5 × 10^5^ murine HSPCs or 1 × 10^5^ (limiting conditions) or 2.5 × 10^5^ (non-limiting conditions) human HSPCs (after *in vitro* priming) or total BM (2.5 × 10^6^) of xenotransplanted mice were injected via the tail vein into recipient mice. Prior to transplantation and for the following 10 days, recipient mice were injected subcutaneously (s.c.) with treprostinil (0.15 mg kg^−1^ 8 h^−1^), cinacalcet (30 mg kg^−1^ 24 h^−1^), or the combination thereof in a total volume of 0.1 mL. In the sequential regimen, recipient mice received HSPCs that had been primed with either treprostinil/forskolin or with cinacalcet and were administered cinacalcet (s.c. or orally; 30 or 10 mg kg^−1^ 24 h^−1^, Seq1) or treprostinil (0.15 mg kg^−1^ 8 h^−1^, Seq2), respectively, *in vivo*. Control mice received unprimed cells and sham-injections of PBS or orally placebo treatment. The *in vivo* doses were selected on the basis of the approved clinical indications of treprostinil and cinacalcet: human doses were converted to their murine equivalent by allometric correction (Reagan-Shaw et al., 2008). Untreated control mice received sham-injections of equal volume.

The experiment was performed in a blinded fashion: the person who injected the drugs or vehicle did not prepare the syringes and was blinded to the content. All animals were monitored daily, and pain and severity assessments were conducted via score sheets according to FELASA recommendations for mice. The overall score defined the intervals of health checks, ranging from daily to twice a day to three times a day, and finally to the humane end point. Briefly, scores were collected by monitoring changes in grimace scales, activity, grooming, food and water intake, breathing, and weight loss. Mice meeting end point criteria were immediately sacrificed by cervical dislocation.

Recovery of peripheral blood cell counts of surviving recipient mice was assessed by quantifying platelets (PLT), granulocytes (GRA) and red blood cells (RBC) using a Hemavet 950FS. For blood collection the “Recommendations for blood sampling in laboratory animals, especially small laboratory animals” by the specialist information from the Committee for Animal Welfare Officers (GV-SOLAS) and Working Group 4 in the TVT; July 2017 were followed. In brief, the vena facialis was punctured using a 4–5.5 mm lancet. The puncture was performed 3–4 mm dorsocaudal to the whorl of hair at the mandible. For repeated blood sampling we followed the rule to not exceed a volume of 1% of the total blood volume per day. To reduce distress, we doubled the group size to bleed each mouse only once a week to obtain blood results of the same experimental group twice a week, respectively.

### 2.11 Homing of murine HSPCs

For homing assays, murine HSPCs were isolated from CD45.1^+^ B6.SJL-PtrcAPep3B/BoyJ donor mice as described in (Kazemi et al., 2016). CD45.2^+^ C57BL6/J recipient mice were lethally (9 Gy) irradiated. After 24 h, they were administered treprostinil (0.15 mg kg^−1^), cinacalcet (30 mg kg^−1^), or the combination thereof subcutaneously in a total volume of 0.1 mL. Subsequently, 2 × 10^5^ primed CD45.1^+^ HSPCs were injected via the tail vein. Untreated control mice received sham-injections of equal volume. After 16 h, bone marrow cells of recipient mice were isolated, and homing was quantified by flow cytometry according to CD45.1 and CD45.2 surface expression.

### 2.12 Platelet activation

16 weeks post transplantation, heparinized blood was collected, and platelet activation was measured by incubating for 15 min with either PBS (control) or ADP (concentration as indicated in Supplmentary Figure S1). In brief, samples were FACS stained according to manufacturer’s guide using anti-human CD61-PB450, anti-mouse CD41-APC, anti-human CD62P-Violet-610, and anti-mouse CD62P-PC7, respectively. Samples were fixed with 2% paraformaldehyde. After erythrocyte lysis, samples were resuspended in 200 µL PBS, and 30.000 PLTs were measured using a CytoFLEX S (Beckman Coulter).

### 2.13 Flow cytometry

Harvested cells (mBM, spleen, blood, CB) were resuspended in FACS buffer. Cell concentration was determined. Cells were washed and stained according to manufacturer’s recommendations. The following antibodies were used and all were purchased from BioLegends: anti-mouse panCD45 (PB), anti-human lineage cocktail (FITC), anti-human panCD45 (FITC), anti-human CD56 (APC), anti-human CD3 (PE), anti-human CD11c (PE-Cy7), anti-human CD19 (BV605), anti-human CD11b (APC-Cy7), anti-human CD34 (APC), anti-human CD38 (PE-Cy7), anti-human CD45RA (BV605), anti-human CD90 (PE), anti-human CD10 (APC-Cy7), anti-human CD49f (PerCP-Cy5.5). Data were acquired using a CytoFLEX S (Beckman Coulter) and analyzed with CytExpert.

### 2.14 Statistical analysis

All data were analyzed using GraphPad Prism or SigmaPlot software. Sample-size calculations were made using R and GINGER Tool. We calculated within a power of 90% to compare all groups with each other, including a control group or against a control group. Differences in survival are visualized by Kaplan-Meier plots and analyzed using the Mantel-Cox log-rank test. Statistical significance of differences between two samples was tested using an unpaired t-test. If more than two data sets were compared, statistical significance was assessed by one-way ANOVA followed by the appropriate *post hoc*-tests (Bonferroni, Dunnett’s or Tukey’s, as indicated in the figure legend).

## 3 Results

### 3.1 Ca^2+^ sensitive adenylyl cyclases are expressed in human HSPCs, but cinacalcet does not inhibit cAMP accumulation in treprostinil/forskolin-treated human HSPCs

**FIGURE 1:**
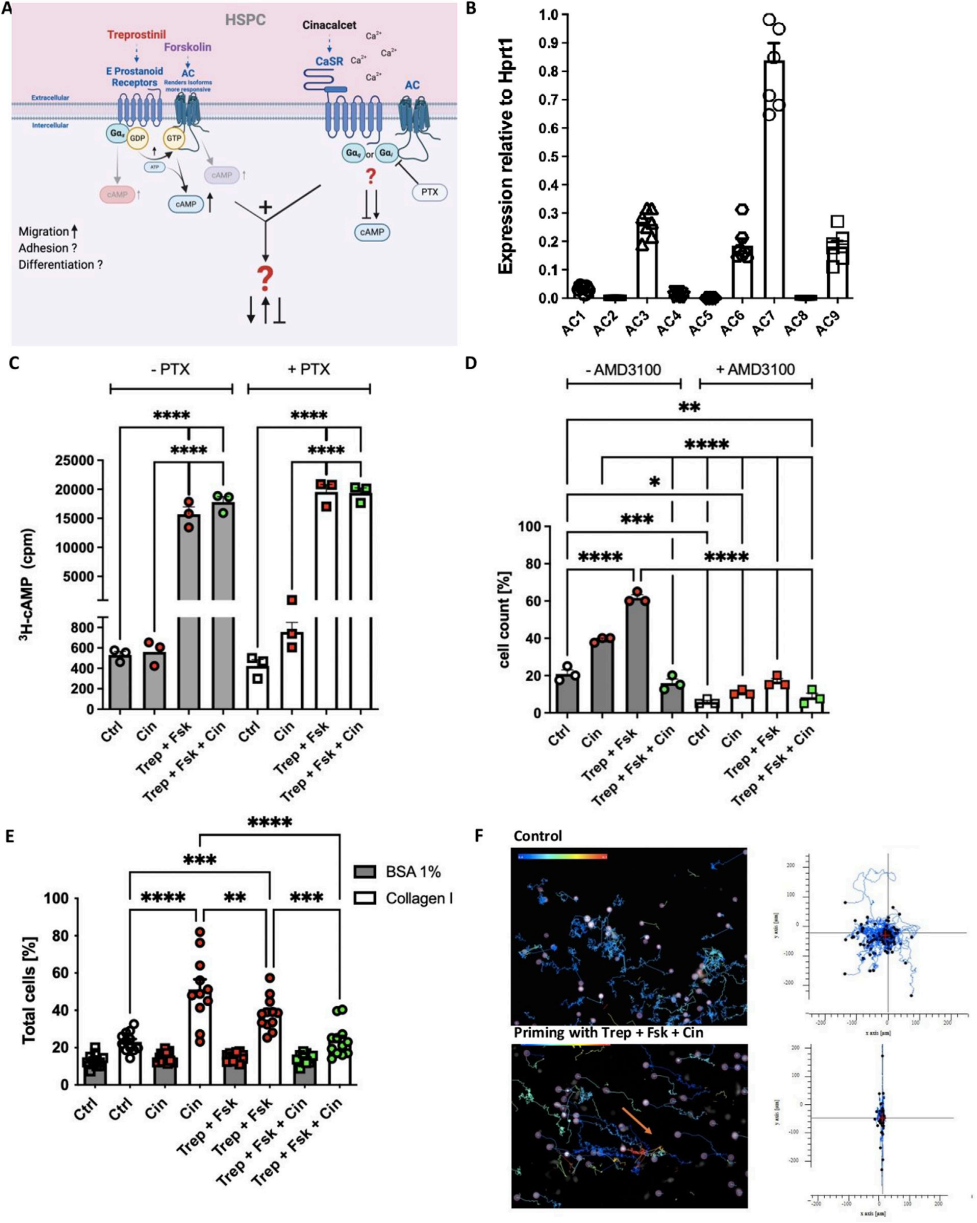
Concomitant stimulation of human HSPCs with treprostinil/forskolin and cinacalcet does not alter the cAMP response but exerts mutual antagonism on their migration and adhesion. **(A)** Schematic illustration (created with BioRender) of GPCR signaling downstream of treprostinil and cinacalcet in human HSPCs. **(B)** Expression of AC isoforms by human HSPCs assessed by qPCR relative to *HPRT*. Data are shown as means ± standard error of the mean (n = 7 donors) **(C)** Human HSPCs were primed for 1 h at 37°C in their respective culture medium in the absence (untreated controls) or in the presence of 2.5 µM cinacalcet, or of 10 µM treprostinil and 30 µM forskolin, or the concomitant combination of 2.5 µM cinacalcet, 10 µM treprostinil and 30 µM forskolin. The cAMP response of primed or unprimed hHSPCs was assessed, with and without pre-incubation with PTX (100 ng mL^−1^) for 16 h (Ctrl; control). The statistical comparison was done by ordinary one-way ANOVA followed by Tukey’s multiple comparison test (n = 3 donors, 1 donor/experiment, 3 independent experiments). **(D)** Migration of primed or unprimed hHSPCs, with and without AMD3100. The statistical comparison was done by ordinary one-way ANOVA followed by Tukey’s multiple comparison test (n = 3 donors, 1 donor/experiment, 3 independent experiments). **(E)** Adhesion of primed or unprimed hHSPCs to collagen type I. The statistical comparison was done by ordinary one-way ANOVA followed by Tukey’s multiple comparison test (n = 6 donors). **(F)** Single-cell movement of unprimed hHSPCs and hHSPCs after priming in the concomitant combination of 2.5 µM cinacalcet, 10 µM treprostinil and 30 µM forskolin, on collagen type I. The tracking diagrams are based on time-lapse imaging (images captured every minute over 7 h) and depict the trajectories of individual human HSPCs towards human SDF-1. The arrows point to where the cells moved.

The interplay between Ca^2+^ and cAMP signaling is fundamental to numerous aspects of cellular functions, including cell migration and cytokine release. The nine isoforms of membrane-bound adenylyl cyclase are expressed in a cell type-specific manner (Ostrom et al., 2022). However, it is not known which isoforms are expressed in human HSPCs. We therefore catalogued human HSPCs for the mRNA expression of AC isoforms by quantitative qPCR. We identified AC3, AC6, AC7 and AC9 as the most abundant isoforms in human HSPCs (Figure 1B). AC3 and AC6 are activated and inhibited, respectively, by Ca^2+^ and protein kinase C; AC6 and AC7 are subject to direct inhibition by G_i_ and AC3 to that by G_βγ_-subunits (Ostrom et al., 2022). Thus, based on the isoforms present in HSPCs, it is conceivable that the cAMP response may be affected by different mechanisms of cross-talk resulting from concomitant stimulation of G_s_-coupled prostanoid receptors and of the G_i_/G_q_-coupled CaSR by treprostinil and cinacalcet, respectively (Figure 1A). We examined a possible crosstalk by determining intracellular cAMP accumulation in human HSPCs by comparing the effect of treprostinil and cinacalcet and their combination. We used forskolin to amplify the cAMP response to treprostinil (Kazemi et al., 2016; Ostrom et al., 2022). Additionally, we preincubated HSPCs in the presence of pertussis toxin to disrupt coupling to G_i_/G_o_ (Kazemi et al., 2016). As illustrated in Figure 1C, cinacalcet *per se* did neither alter basal cAMP levels nor did it affect the cAMP response to the combination of treprostinil and forskolin. In addition, pretreatment with pertussis toxin did not result in any appreciable change in cAMP levels (right handset of bars in Figure 1C). Thus, in human HSPCs, no evidence was found for a synergistic or antagonistic modulation of cAMP formation induced by treprostinil-activated G_s_-coupled prostanoid receptors or by the CaSR.

### 3.2 Treprostinil and cinacalcet are mutually antagonistic on migration, adhesion and movement of human HSPCs

We and others have shown that treprostinil and cinacalcet enhance migration and adhesion of murine and human HSPCs in a CXCR-4 dependent manner (Hoggatt et al., 2009; Kazemi et al., 2016; Lam et al., 2011). Accordingly, we compared the effects of treprostinil/forskolin, cinacalcet and the combination thereof on migration (Figure 1D) and adhesion (Figure 1E) of human and murine HSPCs: treprostinil was more effective in stimulating the migration of human HSPCs towards SDF-1 (right handset of bars in Figure 1D). We verified that the chemotactic response was dependent on CXCR4, as migration was substantially reduced in the presence of the CXCR4 antagonist AMD3100 (=plerixafor) (right handset of bars in Figure 1D). Unexpectedly, the concomitant stimulation with treprostinil/forskolin and cinacalcet resulted in mutual antagonism (fourth bar in Figure 1D). Conversely, cinacalcet was more effective than treprostinil in promoting adhesion to collagen type I, but combined application of both drugs again resulted in mutual antagonism (Figure 1E). We further investigated cell migration in time-lapse movies to visualize the effect of the combination of treprostinil/forskolin (Kazemi et al., 2016) and cinacalcet (Lam et al., 2011). Tracking the movement of individual human CD34^+^ HSPCs allowed for generating a two-dimensional map of the distance covered over the incubation time: the trajectories illustrated in the upper panel of Figure 1F show that control (i.e., untreated) HSPCs underwent large random migration. HSPCs, which had been exposed to the combination of treprostinil/forskolin and cinacalcet, were strikingly different, as their random migration was essentially abolished (lower panel in Figure 1F). Taken together, these observations unequivocally show that simultaneous stimulation of HSPCs by treprostinil and cinacalcet results in mutual antagonism.

### 3.3 Sequential application of treprostinil and cinacalcet enhances survival

During engraftment HSPCs must migrate into and adhere to the bone marrow niche. Thus, their enhanced migration and adherence in response to treprostinil or cinacalcet is likely beneficial for bone marrow reconstitution. Conversely, the mutual antagonism, which we observed *in vitro*, predicts that the signaling pathways stimulated by treprostinil and cinacalcet must not be simultaneously activated during engraftment. Accordingly, we explored, if the sequential application of these drugs improved the outcome of HCT. We resorted to a murine model, which we had previously established to mimic engraftment failure after transplantation of insufficient numbers of HSPCs (Kazemi et al., 2016).

**FIGURE 2:**
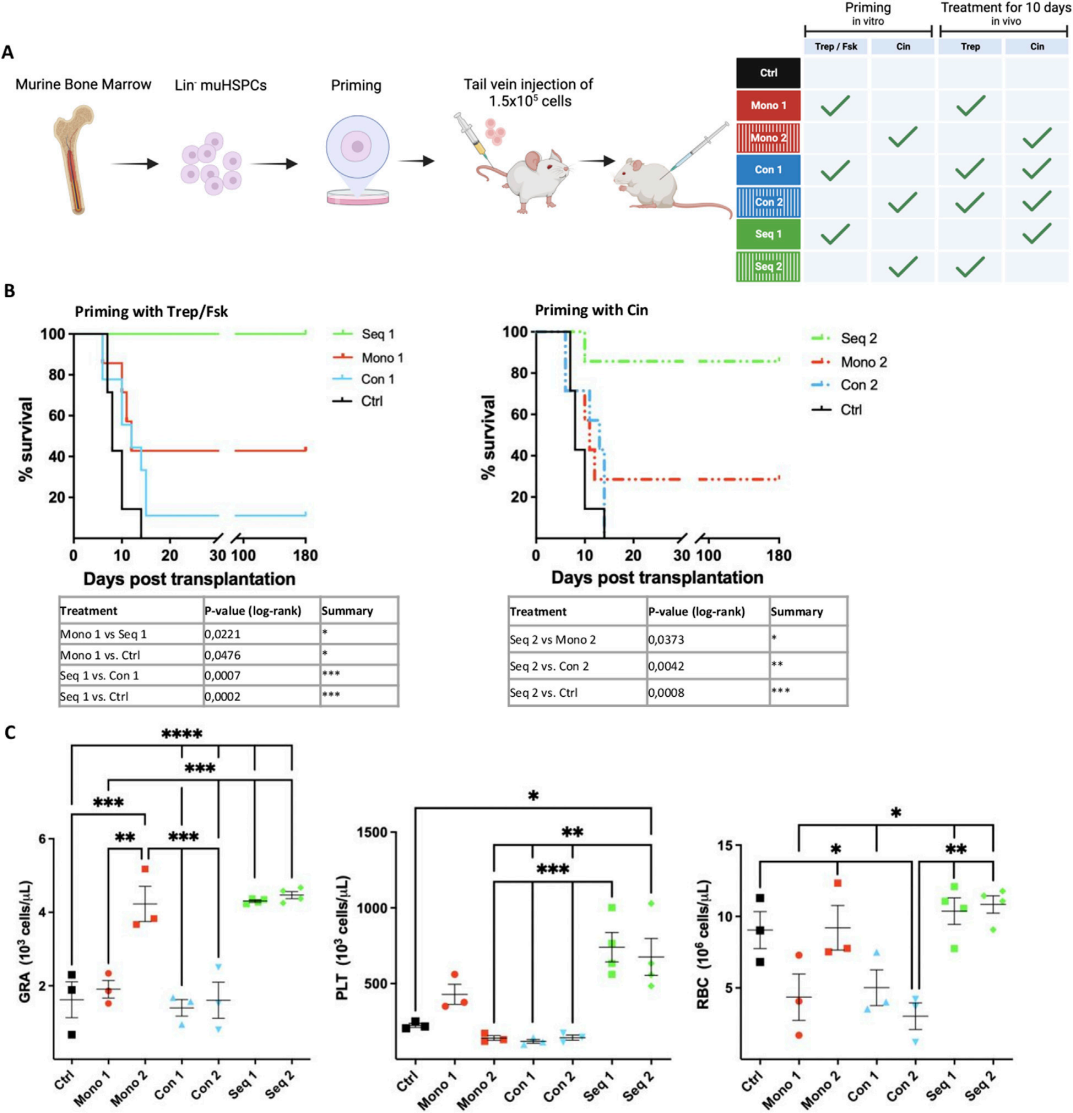
Synergism and mutual antagonism of treprostinil and cinacalcet in rescuing lethally irradiated recipient mice by transplantation of murine HSPCs. **(A)** Outline of the experimental strategy. The scheme was drawn using BioRender. Lethally irradiated recipient mice were injected 1.5 × 10^5^ primed murine HSPCs and administered treprostinil and/or cinacalcet for 10 days. Control mice received unprimed murine HSPCs and sham-injections of PBS. Mice were randomized into 7 groups as indicated in the table. **(B)** Kaplan-Meier curves of survival. Statistical comparison was done by a log-rank test. Illustrated are significantly different results (*P < 0.05; **P < 0.01; ***P < 0.001) (n = 7 mice/group, 49 mice in total). Note that the control curve is the same for the left- and right-hand panel. It is shown twice for illustrative purposes. **(C)** Granulocytes (GRA), platelets (PLT) and red blood cell (RBC) counts of surviving recipient mice assessed on day 7 after transplantation. The statistical comparison was done by one-way ANOVA followed by Tukey’s multiple comparison test multiple comparison *post hoc* test (*P < 0.05; **P < 0.01; ***P < 0.001) (n = 3-4/per group).

As illustrated in Figure 2A, murine BM HSPCs were either primed *in vitro* with treprostinil/forskolin and recipient animals were then administered cinacalcet (regimen referred to as Seq 1), or *vice versa* priming was done *in vitro* with cinacalcet and recipient animals received treprostinil (regimen Seq 2). The outcomes of these regimens were compared to those observed with (i) HSPCs primed with either treprostinil/forskolin or cinacalcet *in vitro* and concomitant *in vivo* administration of the combination of treprostinil and cinacalcet to recipient animals (regimens Con 1 and Con 2), with (ii) HSPCs and recipient animals exposed solely to each single agent (regimens Mono 1 and Mono 2), and (iii) to that seen in recipient animals receiving untreated HSPCs (Ctrl). The limiting number of murine BM-derived HSPCs (1.5 × 10^5^) did not suffice to rescue the control group of lethally irradiated animals (Figure 2B, black lines). Treatment with single agents (Mono 1 and Mono 2) afforded partial rescue (Figure 2B, red lines). In agreement with the mutual antagonism observed *in vitro*, the survival rate of recipient mice was not improved upon concomitant *in vivo* administration of treprostinil and cinacalcet (Figure 2B, blue lines Con 1 and Con 2). Most importantly, the sequential regimen (i.e., Seq 1 and Seq 2) substantially improved the survival of recipient mice (Figure 2B, green lines). Finally, it is worth noting that in all scenarios tested, *in vitro* priming of HSPCs with treprostinil/forskolin (Figure 2B, left-hand panel; Mono 1, Seq 1, Con 1) consistently resulted in better survival than priming with cinacalcet (Figure 2B, right-hand panel; Mono 2, Seq 2, Con 2).

In line with improved survival, peripheral blood cell recovery was significantly better in mice receiving the Seq 1 or Seq 2 regimen: 7 days post-transplantation, granulocyte (GRA) and platelet (PLT) counts were significantly higher in these mice than those seen in control mice and in mice allocated to the concomitant regimen Con 1 and Con 2 groups (Figure 2C).

Granulocyte recovery was also enhanced in mice treated solely with cinacalcet (Mono 2), but not in mice subjected to the other regimens (Mono 1, Con 1 or Con 2). Hence, monotherapy with cinacalcet sufficed to accelerate granulocyte recovery, but the sequential combination was superior because of the additional enhancement of platelet recovery. The mutual antagonism of concomitant cinacalcet and treprostinil was also evident from the impaired recovery of granulocyte and platelet counts, which presumably reflected the reduced capacity of transplanted HSPCs to repopulate the bone marrow.

### 3.4 Regimen Seq 1 promotes the early onset and rapid increase of granulocyte and platelet recovery in peripheral blood

**FIGURE 3:**
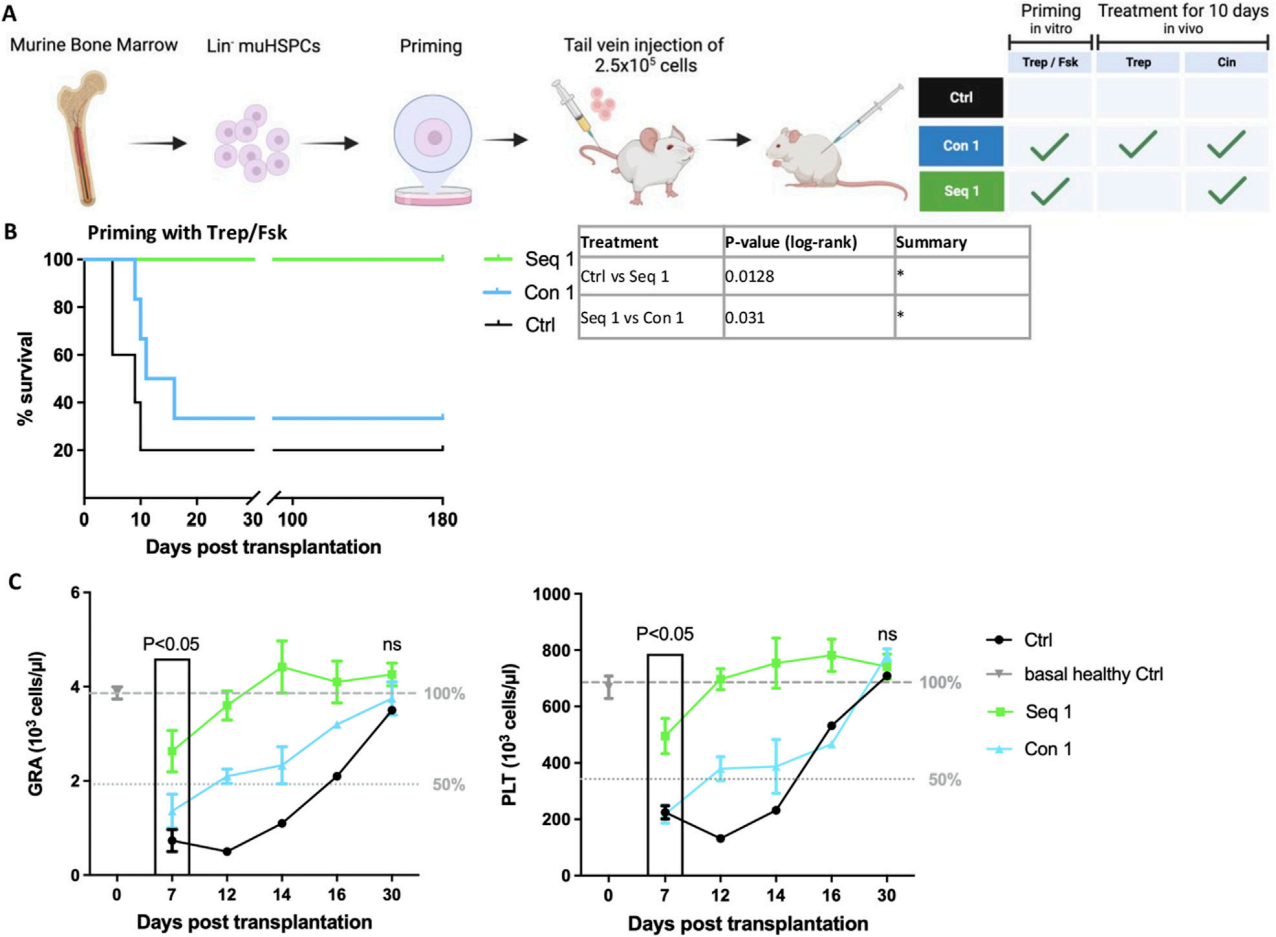
Sequential regimen of mHSPCs priming with treprostinil/forskolin and cinacalcet-administration (Seq 1) accelerates recovery of peripheral blood in murine HCT. **(A)** Outline (drawn with BioRender) of the experimental strategy: lethally irradiated recipient mice were injected 2.5 × 10^5^ murine HSPCs, which had been primed with and Con1 and Seq 1 *in vivo* treatments. Control mice received mHSPCs without priming and sham-injections of PBS. Mice were randomized into 3 groups as indicated in the table. **(B)** Kaplan-Meier curves of survival. Statistical comparison was done by a log-rank test. (*P < 0.05; **P < 0.01; ***P < 0.001; (n = 5 mice/Ctrl and Seq 1, n = 6 mice/Con1, 16 mice in total). **(C)** Granulocyte (GRA) and platelet (PLT) counts of recipient mice assessed over time as indicated. The dotted and dashed lines indicate 50% and 100%, respectively, of average cell counts seen in healthy control animals. An unpaired two-tailed t-test was used to assess the difference between animals subjected to the Seq 1 and the Con 1 regimen for statistical significance. Error bars represent means ± SEM.

The data summarized above highlighted the difference between sequential and concomitant regimen, which resulted in synergism and mutual antagonism, respectively. Because of the low number of surviving animals, it was not possible to assess the time course of granulocyte and platelet recovery in a comparative manner. Accordingly, we repeated the experiment with a higher cell dose (2.5 × 10^5^ HSPCs/recipient animal) and monitored platelet and granulocytes counts for up to 30 days after transplantation. We focused on regimen Seq 1, i.e., *in vitro* priming of HSPCs with treprostinil/forskolin and administration of cinacalcet *in vivo* (Figure 3A), because this was the most effective regimen (*cf*. Figure 2B). Again, the mutual antagonism of treprostinil and cinacalcet was evident: all recipient mice subjected to the Seq 1 regimen survived (Figure 3B, green line), but the majority of recipient mice, which had been administered the combination of cinacalcet and treprostinil (Con 1) succumbed to bone marrow failure (Figure 3B, blue line). In fact, survival of mice subjected to the Con 1 regimen was only marginally improved over that of control animals, which had been injected unprimed HSPCs and were subsequently administered vehicle injections (Figure 3B, black line).

Platelets and granulocytes were counted in peripheral blood obtained from the surviving mice. In recipient mice subjected to the Seq 1 regimen (Figure 3C, green symbols), recovery of granulocytes and platelets was significantly more rapid compared to mice, which were concomitantly administered treprostinil and cinacalcet up to day 16 (Figure 3C, blue symbols). Similarly, recovery of granulocytes and platelets was delayed in the single surviving mouse of the control group (Figure 3C, black symbols). It should be noted, that after 7 days, only one surviving mouse was recorded. On day 30, all surviving animals had comparable counts of platelets and granulocytes. In fact, they were within the normal range indicating effective reconstitution of hematopoiesis. These findings show that a regimen based on the sequential application of treprostinil and cinacalcet has translational potential in preventing graft failure, as it promotes the rapid recovery of granulocytes and platelets after HCT. Given that lower doses of HSPCs with enhanced potency are necessary for efficient engraftment, the sequential treatment described herein shows promise in reducing the required cell number. Consequently, this approach may alleviate the burden and risks associated with human HSPC donations.

### 3.5 Treprostinil treatment promotes HSPC homing while Seq 1 treatment fosters rare HSPC populations

**FIGURE 4:**
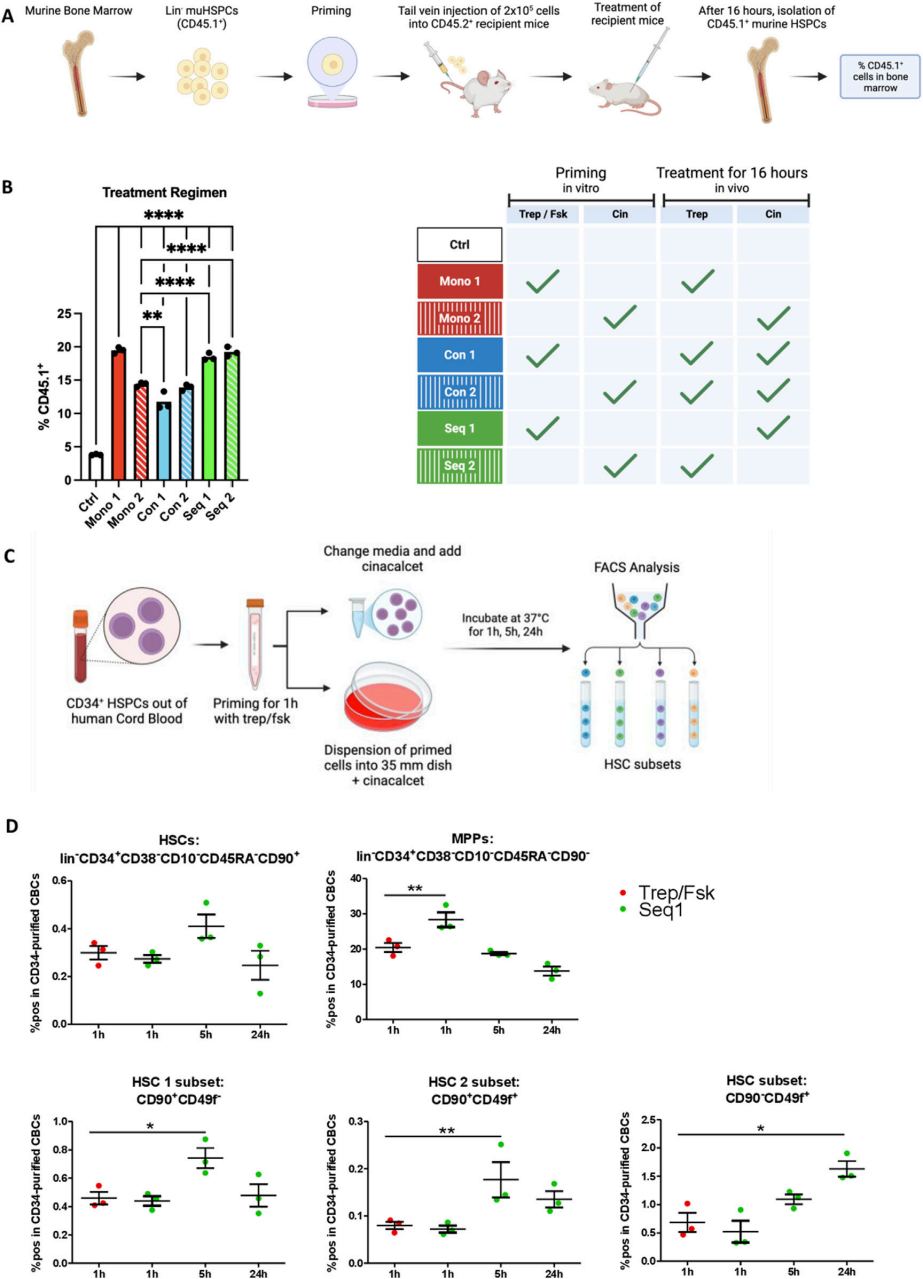
Homing of HSPCs treated with treprostinil is enhanced while Seq 1 treatment promotes rare HSPC populations. **(A)** Outline of the experimental approach (drawn with BioRender): CD45.1^+^ mHSPCs isolated from bone marrow of B6.SJL-PtrcAPep3B/BoyJ donor mice were left unprimed (control, Ctrl) or primed *in vitro* for 1 h as indicated and were subsequently transplanted into recipient CD45.2^+^ C57BL/6J mice (2 × 10^5^ cells/mouse) via tail vein injection. Mice were randomized into 7 groups as indicated in the table (n = 3 mice/group, 21 mice in total). **(B)** 16 h after HSPC transplantation, bone marrow cells were harvested from femora of recipients, immunostained for CD45.1 and CD45.2 and their relative proportion quantified by flow cytometry. The statistical comparison was done by one-way ANOVA followed by Tukey’s multiple comparison test. Significantly different results are illustrated as: *P < 0.05; **P < 0.0021; ***P < 0.0002; ****P< 0.0001. **(C)** Outline of the experimental approach (drawn with BioRender): human HSPCs were primed with treprostinil/forskolin for 1 h. Subsequently, they were washed and resuspended in medium containing growth factors in the presence of cinacalcet. After 1 h, 5 h and 24 h, cells were collected and HSCs subsets were analyzed using FACS. For the 24 h’ time point, cells were resuspended in methylcellulose medium containing growth factors required for supporting differentiation. **(D)** Percentage of positive cells in CD34-purified CB HSPCs of a set of HSC subset markers (n = 3; technical triplicates). The statistical comparison was done by one-way ANOVA followed by Dunnett’s multiple comparison test. Significantly different results are illustrated as: *P < 0.05; **P < 0.01; ***P < 0.001.

After intravenous injection, homing of HSPCs is the first step required in bone marrow reconstitution. We assessed the effects of the different treatment regimens on homing of murine HSPCs by priming CD45.1^+^ HSPCs and subsequently injecting them into CD45.2^+^ recipient mice. Recipient mice were also administered treprostinil and/or cinacalcet. The control was the absence of any treatment, i.e., injection of unprimed CD45.1^+^ HSPCs and subsequent vehicle administration *in vivo*. After 16 h, HSPCs were retrieved from the bone marrow and quantified by flow cytometry (Figure 4A). It is evident from Figure 4B that the highest number of CD45.1^+^ HSPCs was retrieved from the bone marrow of recipient mice, which had been subjected to regimens Seq 1, Seq 2 and Mono 1. In contrast, the bone marrow of animals subjected to the regimen Mono 2 (i.e., priming with cinacalcet and subsequent administration of cinacalcet) contained less CD45.1^+^ HSPCs (Figure 4B, third bar). Finally, concomitant administration of treprostinil and cinacalcet again resulted in mutual antagonism, because the number of CD45.1^+^ HSPCs was lower in the regimens Con 1 and Con 2 compared to that observed in Seq 1 and Seq 2 (Figure 4B). We know that pretreating cells with treprostinil/forskolin does not alter apoptosis or cells cycle (Kazemi et al., 2016). However, we cannot fully exclude that this increase of CD45.1^+^ HSPCs could also result from increased cell proliferation or reduced cell death. Taken together, these observations indicated that treprostinil was more effective in supporting homing than cinacalcet, but that homing did not *per se* suffice to account for enhanced survival in the regimen, since survival rates within the regimen Mono 1 were lower than in the regimen Seq 1 and Seq 2 (*cf*. Figure 2B).

Rare Hematopoietic stem cells (HSCs) isolated from UCB have been characterized by distinct surface marker profiles (Anjos-Afonso et al., 2022), including CD45^+^CD34^+^CD38^−^CD45RA^−^CD90^+^CD49f^−^ (HSC1 subset) and CD45^+^CD34^+^CD38^−^CD45RA^−^CD90^+^CD49f^+^ (HSC2 subset). RNA sequencing (RNA-Seq) analysis revealed that not only the CD90^+^CD49f^+^ subset but also the CD90^−^CD49f^+^ subset harbors self-renewing HSCs. Both CD49f^+^ subpopulations exhibit the highest repopulation potential when xenotransplanted into NSG mice. To investigate whether Seq 1 treatment modulates the composition of rare HSC subsets, CD34^+^ cells were treprostinil/forskolin primed following cinacalcet treatment for various time points and the stem cell populations were characterized using flow cytometry (Figure 4C). After 1 hour of treatment, no significant differences were observed compared to the control group, except for an increase in multipotent progenitors (MPPs). However, after 5 h of treatment, all HSC populations, including HSC1 and HSC2, showed a significant increase. Notably, the single CD49f^+^ HSC subset was markedly elevated 24 h post-treatment (Figure 4D).

Collectively, these findings suggest that *in vitro* pretreatment with treprostinil/forskolin primarily promotes rapid homing, whereas cinacalcet modulates the stem cell pool, particularly enhancing the CD49f^+^ HSC subsets, which support long-term engraftment.

### 3.6 Cinacalcet synergizes with priming of HSPCs by treprostinil/forskolin in promoting outgrowth of CFU-GEMMs

**FIGURE 5:**
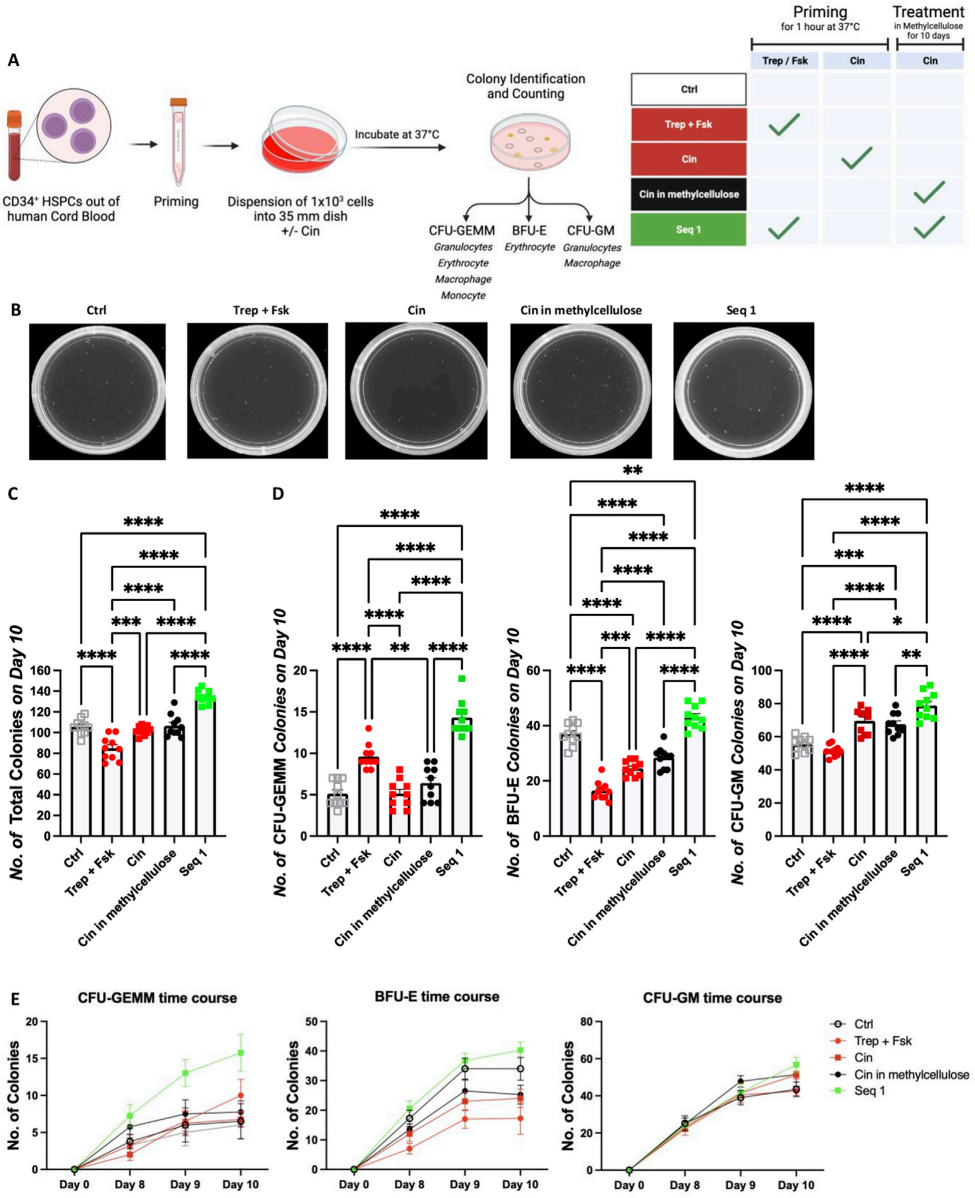
Priming by treprostinil/forskolin and propagation with cinacalcet synergistically promote colony formation by human CD34^+^ HSPCs. **(A)** Outline of the experimental approach (drawn with BioRender): human HSPCs were left unprimed or primed with treprostinil/forskolin or cinacalcet. Subsequently, they were resuspended in methylcellulose medium containing growth factors required for supporting differentiation for 10 days in the absence or presence of cinacalcet. **(B)** Representative photographs of colonies taken on day 10. The colonies were identified according to size and morphology and counted. **(C)** Total number of all colonies, **(D)** number of CFU-GEMM, BFU-E and CFU-GM colonies counted on day 10. The statistical comparison was done by one-way ANOVA followed by Tukey’s multiple comparison (*P < 0.03; **P < 0.002; ***P < 0.0002; ****P < 0.00001, n = 5 independent donors in duplicates). **(E)** Colony numbers counted on days 8, 9 and 10 (depicted are all 5 donors in duplicates over time, ±SD).

After reaching the bone marrow niche, HSPCs must undergo differentiation to reconstitute hematopoiesis. We posited that the efficacy of regimen Seq 1 resulted from synergism, where the subsequent action of cinacalcet promoted differentiation of HSPCs primed by treprostinil/forskolin. This hypothesis was examined in colony forming unit assays (CFU) in methylcellulose: because of their translational relevance, we focused on human CD34^+^ HSPCs and compared the effect of cinacalcet on unprimed HSPCs versus treprostinil/forskolin-primed HSPCs (Figure 5A). We counted colonies comprising all lineages, including those representing the earliest progenitors i.e., multi-lineage CFU-GEMMs (granulocyte erythrocyte macrophage megakaryocyte), BFU-Es (burst forming unit erythroid and CFU-GM (granulocyte macrophage). After 10 days, we observed that priming with treprostinil/forskolin and subsequent culture in methylcellulose containing cinacalcet (equivalent to the regimen Seq 1) was most effective in enhancing total colony formation as well as all three distinct colony types (Figures 5B, C).

When examining individual types of colonies, we found that under this condition, the number of multi-lineage CFU-GEMMs was increased compared to all other conditions (Figure 5D, left panel). This difference was already evident on days 8 and 9 (Figure 5E, left hand panel). Interestingly, both priming with treprostinil/forskolin and with cinacalcet impaired the formation of BFU-Es. Moreover, the number of BFU-Es was also lower in unprimed HSPCs maintained in the presence of cinacalcet than in the unprimed control HSPCs. Formation of BFU-Es was, however, restored under the culture condition corresponding to Seq 1 (middle panels in Figures 5D, E). This indicated that treprostinil, in particular, had an inhibitory action on the erythroid lineage, a finding consistent with the reduction in red blood cell content 10 days after transplantation (cf. regimen Mono 1 in Figure 2C, right hand panel). Sole priming of HSPCs with treprostinil/forskolin did not affect the differentiation of HSPCs into the granulocyte/macrophage lineage (right hand panels in Figures 5D, E). However, both priming of HSPCs with cinacalcet or their maintenance in cinacalcet stimulated the outgrowth of CFU-GM slightly less than seen in the condition corresponding to Seq 1 (right hand panels in Figure 5D.

In conclusion, the findings illustrated in Figures 4, 5 indicate that treprostinil, forskolin, and cinacalcet operate through distinct mechanisms. Specifically, treprostinil promotes homing efficiency, whereas Seq 1 selectively amplifies rare HSC populations, which are essential for successful engraftment and differentiation. To translate these insights into clinical applications, we evaluated the synergistic effects on recipient survival in xenotransplantation models.

### 3.7 Seq 1 promotes engraftment of xenotransplanted human HSPCs

**FIGURE 6:**
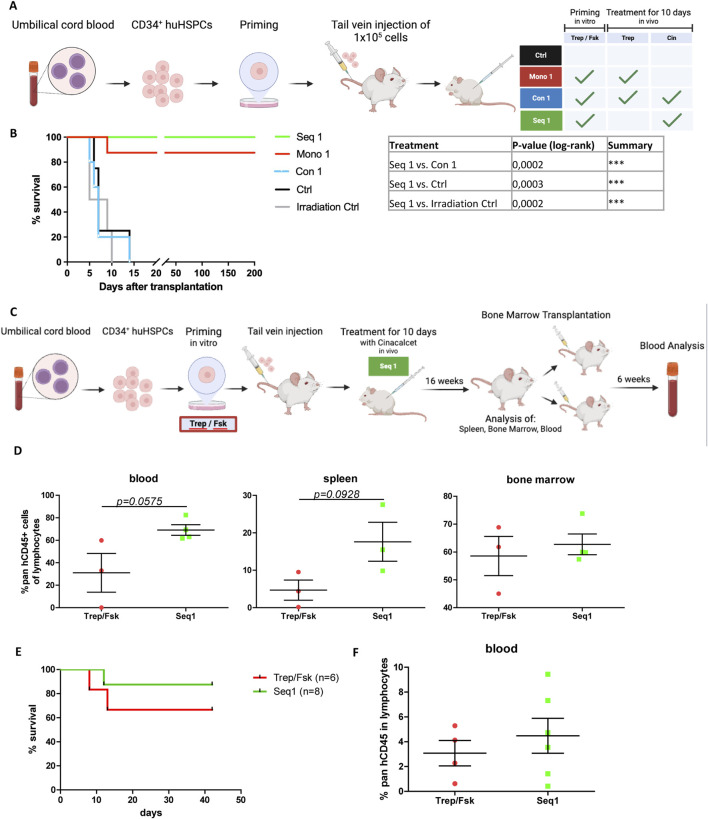
Rescue of xenotransplanted NSG recipient mice by sequential priming of human HSPCs with treprositinil/forskolin and cinacalcet administration. **(A)** Outline of the experimental approach (drawn with BioRender): human HSPCs were primed or left unprimed for 1 h with treprostinil/forskolin. 1.5 × 10^5^ primed or non-primed human HSPCs were injected, and mice were subjected to *in vivo* treatment. Control mice received human HSPCs without priming and sham-injections of PBS. Mice were randomized into 4 treatment groups as indicated in the table (n = 5 mice/group, n = 4 in the irradiation group, 24 mice in total). **(B)** Kaplan-Meier curves of survival. The statistical comparison was done with a log-rank test (***P < 0.001). **(C)** Schematic overview of the experimental design (created with BioRender). Human HSPCs were primed with treprostinil and forskolin for 1 h, followed by the injection of 2.5 × 10^5^ primed HSPCs into mice. The mice were then treated *in vivo* with cinacalcet for 10 days (oral, 10 mg kg^−1^ 24^−1^). After 16 weeks, spleen, bone marrow, and blood samples were collected and analyzed. Bone marrow cells were subsequently transplanted into two additional mice for a second transplant, and blood analysis was performed 6 weeks later. Control mice received primed HSPCs followed by placebo treatment. Mice were randomized into treatment groups for the first (n = 3 in control group, n = 4 in treatment group, 7 mice in total) and second (n = 6 in control group, n = 8 in treatment group, 14 mice total) transplant. **(D)** FACS analysis of blood samples following the first transplant, showing the percentage of human CD45^+^ cells within the lymphocyte population. Statistic comparisons were performed using an unpaired t-test **(E)** Kaplan-Meier survival curves of the second transplant. Statistical comparisons were performed using a log-rank test. **(F)** FACS analysis of blood samples following the second transplant, showing the percentage of human CD45^+^ cells within the lymphocyte population. Statistic comparisons were performed using an unpaired t-test.

The regimen Seq 1 is of potential relevance to overcome limitations in human patients undergoing cord blood HCT (Gupta and Wagner, 2020). We further verified the translational implications by xenotransplantating human CB-derived CD34^+^ HSPCs into NSG recipient mice (Figure 6A). A limiting dose of HSPCs was selected to mimic the clinical scenario of patients at high risk of engraftment failure due to the transplantation of insufficient cell doses.

Accordingly, lethally irradiated recipient mice which received unprimed HSPCs, succumbed as rapidly to BM failure as the irradiation control animals (black and grey lines, respectively, in Figure 6B). In contrast, all mice undergoing the regimen Seq 1 were rescued (Figure 6B, green line). In fact, these animals survived for more than 200 days after transplantation. The regimen Mono 1 (i.e., injection of HSPCs primed with treprostinil/forskolin and subsequent administration of treprostinil) achieved a survival rate of 87.5% (Figure 6B, red line), which was consistent with previously published results (Kazemi et al., 2016). Finally, mutual antagonism was again observed: recipient animals subjected to the Con 1 regimen (i.e., injection of HSPCs primed with treprostinil/forskolin and subsequent concomitant administration of cinacalcet and treprostinil) were as likely to die from BM failure as the irradiation control (Figure 6B, blue line).

To evaluate the long-term repopulation potential of Seq 1-treated xenografted CD34^+^ human HSPC in a more clinical setting, we transplanted non-limiting cell numbers into sub-lethally irradiated NSG mice. We then assessed the human lineage compartment 16 weeks post-transplantation, followed by a secondary transplantation (Figure 6C). Treprostinil/forskolin-primed cells served as controls. Consistent with our hypothesis, Seq 1 treatment enhanced survival (Figure 6E). Although not significant but by trend pan-hCD45^+^ cell infiltration in blood, bone marrow, and spleen was better when compared to control animals (Figure 6D; Supplementary Figure S1A).

We also analyzed lineage differentiation and platelet functionality. While platelet dysfunctions have been documented in various disease states (Pihusch et al., 2002; Akahoshi et al., 2016; Mott et al., 2024), Seq 1 treatment did not impair platelet function (Supplementary Figure S1A). Importantly, CD34^+^ HSPCs differentiated into all major lineages, including T cells, NK cells, B cells, and myeloid cells (Supplementary Figures S1B–D). Notably, compared to treprostinil/forskolin-primed controls, Seq 1-treated animals exhibited slightly reduced numbers of CD19^+^ cells (Supplementary Figures S1B–D).

In the secondary transplantation, Seq 1-treated total bone marrow cells showed enhanced repopulation capacity, resulting in a greater number of rescued mice and elevated levels of pan-hCD45^+^ cells (Figure 6F, green line). Six weeks post-transplantation, cells of the human immune compartment were differentiated (Supplementary Figure S1E).

In summary, the Seq 1 treatment regimen not only promotes survival but also enhances the long-term repopulation capacity of HSPCs, supports their differentiation potential, and, to some extent, prevents transplanted HSPCs from exhaustion.

## 4 Discussion

Considering the burden on donors and the limitations associated with PB and BM harvesting in clinical practice (Hequet, 2015; Moalic, 2013; Piccirillo et al., 2023; Prisciandaro et al., 2024; Chen et al., 2013), CB warrants attention as an alternative source of HSPCs. However, its widespread clinical application is hindered by delayed engraftment and incomplete immune reconstitution.

Historically, two main strategies have been pursued to promote CB-based HCT: (i) increasing the dose of HSPCs by their *ex vivo* expansion and (ii) enhancing their homing and subsequent engraftment through *in vitro* incubation with priming compounds (Gupta and Wagner, 2020).

However, only the successful homing of HSCs can ensure the initiation of long-term repopulation of the blood system. Thus, homing and engraftment are critical limiting factors, especially considering that growth factor-expanded HSPCs are less likely to repopulate the BM niche (van der Loo and Ploemacher, 1995). Consequently, overcoming low efficacy of engraftment in clinical HCT has been identified as a significant unmet medical need and as a target in the treatment of previously incurable hematopoietic diseases. We previously demonstrated the efficacy of treprostinil in enhancing HSPC engraftment (Kazemi et al., 2016). Similarly, earlier work showed that the preincubation of HSPCs in the presence of cinacalcet boosted their lodgment in the endosteal niche and their capacity to reconstitute hematopoiesis *in vivo* (Lam et al., 2011). A sequential regimen, where HSPCs were first pretreated with treprostinil/forskolin and recipient mice were then administered therapeutic doses vildagliptin, was superior to a regimen based solely on treprostinil. The synergism of sequential treprostinil and vildagliptin treatment can be rationalized by taking into account that treprostinil enhances CXCR4 expression and vildagliptin inhibits DPP4 (dipeptidyl peptidase-4), which cleaves the CXCR4 cognate agonist SDF-1α/CXCL12 (Zebedin-Brandl et al., 2020). The receptors for treprostinil and cinacalcet operate via distinct signaling pathways (Messa et al., 2008; Chavez-Abiega et al., 2020). It was therefore not clear, if and under which conditions they synergized. In the present study, we addressed this question and explored their combined action. The experiments provided four major *in vitro* insights: (i) Treprostinil was more effective in stimulating migration of human HSPCs than cinacalcet. (ii) Cinacalcet was superior in promoting adhesion. (iii) Treprostinil and cinacalcet were mutually antagonistic, when concomitantly present. (iv) A transient rise of cAMP levels, which was induced through priming with treprostinil/forskolin, sufficed to promote subsequent differentiation of early progenitors (CFU-GEMMs) but impaired the outgrowth of the erythroid lineage (BFU-Es).

**FIGURE 7:**
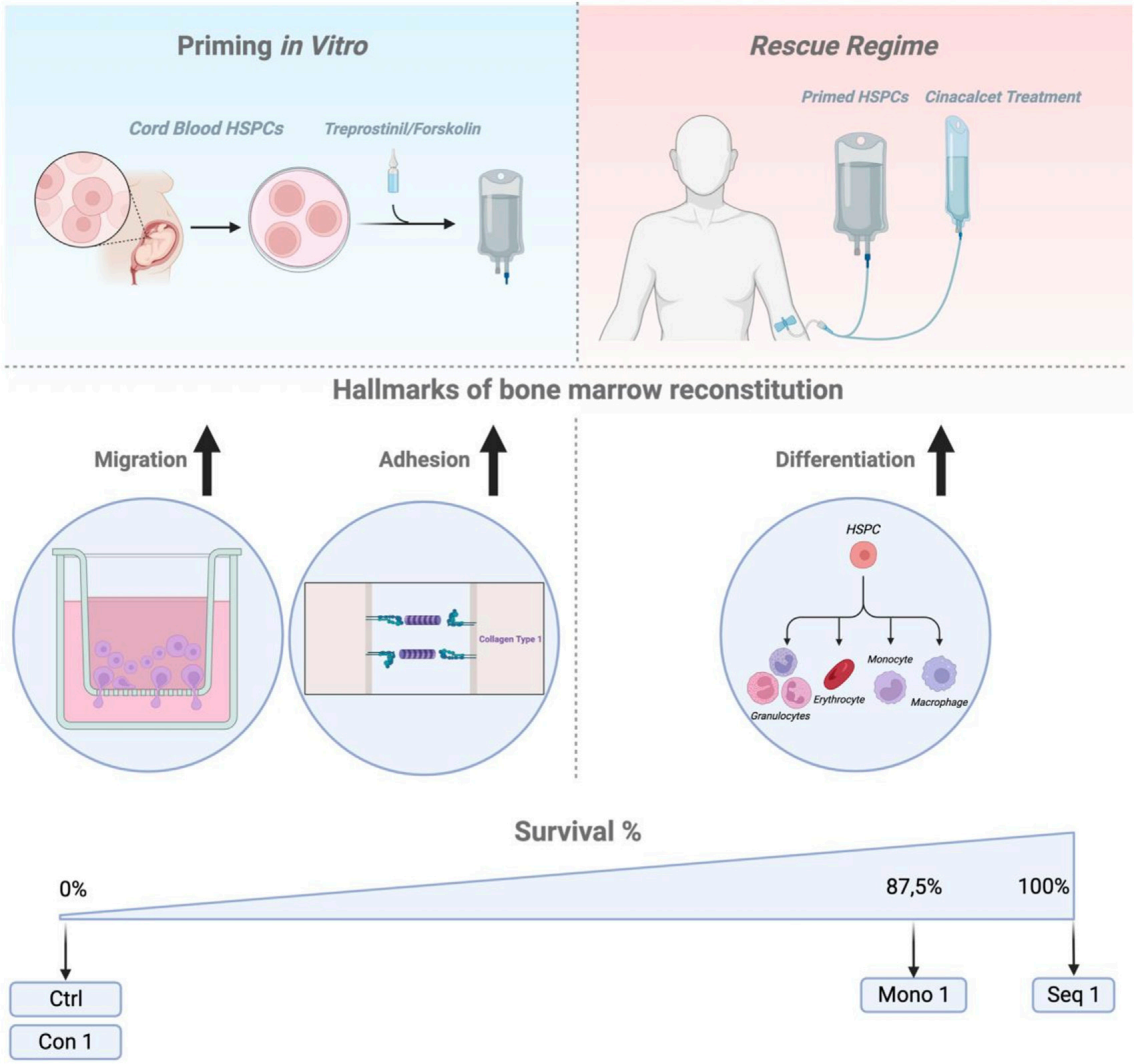
Summary Figure. Schematic representation (drawn with BioRender) summarizing the key findings and outlining a potential application of the Seq 1 as a rescue regimen for patients undergoing cord blood HCT.

These insights allowed us to define an optimal HCT regimen (Figure 7): sequential priming of murine or human HSPCs with treprostinil and forskolin, followed by cinacalcet treatment in recipients. This approach led to faster PB recovery and 100% survival in transplanted mice. The mutual antagonism observed *in vitro* was also confirmed *in vivo* for both murine and human HCTs.

The translational relevance of our findings was confirmed in lethally irradiated mice xenotransplanted with limited CB-derived CD34^+^ HSPCs, where all Seq 1-treated animals survived. This approach also improved human cell engraftment and immune cell differentiation under clinical-like, non-limiting transplantation conditions. Furthermore, Seq 1 treated HSPCs demonstrating enhanced long-term repopulation and, to some extent, protection against HSC exhaustion.

From a clinical perspective, it is important to note that the administration of treprostinil to patients undergoing HCT presents a significant limitation: treprostinil effectively inhibits platelet aggregation (Broxmeyer et al., 2005). This increased risk of bleeding is a major concern in myeloablated patients. Consequently, treprostinil will not be directly applied to patients in Seq 1. It will only be used for priming *in vitro*. We could successfully demonstrate that platelet functions in the presented settings are unaltered.

In contrast, hypocalcemia, the primary risk associated with the administration of cinacalcet, appears manageable by monitoring blood Ca^2+^ levels, the QT-interval in ECG recordings and appropriate supplementation via intravenous administration (Messa et al., 2008).

Based on all these considerations, the Seq 1 regimen seems preferable for translation into clinical applications. Nevertheless, further studies are required to define the minimal required efficacious dose of cinacalcet and robust measures to safeguard clinical trial participants.

Our experiments focused on the cell-autonomous action of treprostinil and cinacalcet. However, the BM niche regulates hematopoietic homeostasis and provides extrinsic cues for HSPCs (Morrison and Scadden, 2014). It is evident that, when administered to recipient animals, both compounds are likely to affect cells residing in the bone marrow niche. This is apparent for treprostinil, as prostaglandin E2 influences the trabecular bone structure (Ostrom et al., 2022), and for cinacalcet, as the CaSR is expressed on osteoblasts and osteoclasts (Anjos-Afonso et al., 2022). Further experiments need to be done to fully understand the mechanisms triggered in stem cells by Seq 1 regimen.

Cell-based therapies hold great promise in clinical medicine, but their development must follow a strict regulatory pathway to ensure the safety and efficacy of advanced therapy medicinal products (ATMPs). While HCT, as a well-established somatic cell therapy (Pushpakom et al., 2019), is exempt from regulatory approval, primed HSPCs must meet safety, efficacy, and manufacturing standards similar to traditional drugs. Establishing preclinical proof-of-concept in animal models remains a key challenge for cell therapy products (Gupta and Wagner, 2020).

Our experiments directly addressed this challenge by adoptively transferring human CB-derived HSPCs into immunodeficient NSG mice (Adams et al., 2006; Whittle et al., 2012). Combined with thorough *in vitro* characterization, this approach effectively bridged the gap between murine BM-derived HSPCs and human CB-derived counterparts. The compounds used are promising candidates for drug repurposing (Pihusch et al., 2002): they have well-characterized human pharmacodynamics, pharmacokinetics, and safety profiles, facilitating clinical translation at lower costs and within a reasonable timeframe (Broxmeyer et al., 2005; Chavez-Abiega et al., 2020).

## 5 Conclusion

Based on our findings, we propose that the sequential use of treprostinil and cinacalcet could revive CB-HCT as the first “rapid engraftment regimen.” This approach has also the potential to enhance the efficacy of all HCTs, regardless of the HSPC source.

By boosting the potency of HSPCs, improving their BM reconstitution potential, and enhancing their homing and engrafting properties both *in vitro* and *in vivo*, this regimen promises two significant advantages: i) It enables successful transplants even with sub-threshold HSPC collections and ii) it reduces the number of cells needed from donors, thereby minimizing the risks and discomfort associated with bone marrow and peripheral stem cell harvesting. This, in turn, increases the pool of willing HSPC donors and allows a broader use of HCT in various clinical settings.

Future studies are ongoing to investigate the safety and precise mechanisms of the Seq 1 regimen. This novel regimen could transform the landscape of HCT, making it safer and more accessible for patients and donors alike.

## Data Availability

The raw data supporting the conclusions of this article will be made available by the authors, without undue reservation.

## Abbreviations

AC, adenylate cyclase; BFU-E, burst forming unit erythroid; cAMP, cyclic adenosine monophosphate; CaSR, calcium-sensing receptor; CB, cord blood; CB-HCT, cord blood hematopoietic cell transplantation; CFU, Colony forming unit; CFU-GEMM, Colony forming unit - granulocyte erythrocyte macrophage megakaryocyte; CFU-GM, Colony forming unit - granulocyte macrophage; Cin, cinacalcet; CXCR4, C-X-C motif chemokine receptor 4; Fsk, forskolin; HCT, hematopoietic cell transplantation; HSC, hematopoietic stem cell; HSPCs, hematopoietic stem and progenitor cells; hHSPCs, human hematopoietic stem and progenitor cells; GPCR, G protein coupled receptor; MACS, magnetic beads cell sorting; MAPK, mitogen-activated protein kinase; mHSPCs, mouse hematopoietic stem and progenitor cells; PTX, pertussis toxin; SDF-1, stromal-derived-factor-1; Trep Treprostinil.
