# Supplementary figures and images for: Repurposing the prostaglandin analogue treprostinil and the calcium-sensing receptor modulator cinacalcet to revive cord blood as an alternate source of hematopoietic stem and progenitor cells for transplantation
Source: Front Pharmacol. 2025 Jan 9;15:1444311. doi: 10.3389/fphar.2024.1444311 (PMC11755040; doi:10.3389/fphar.2024.1444311)

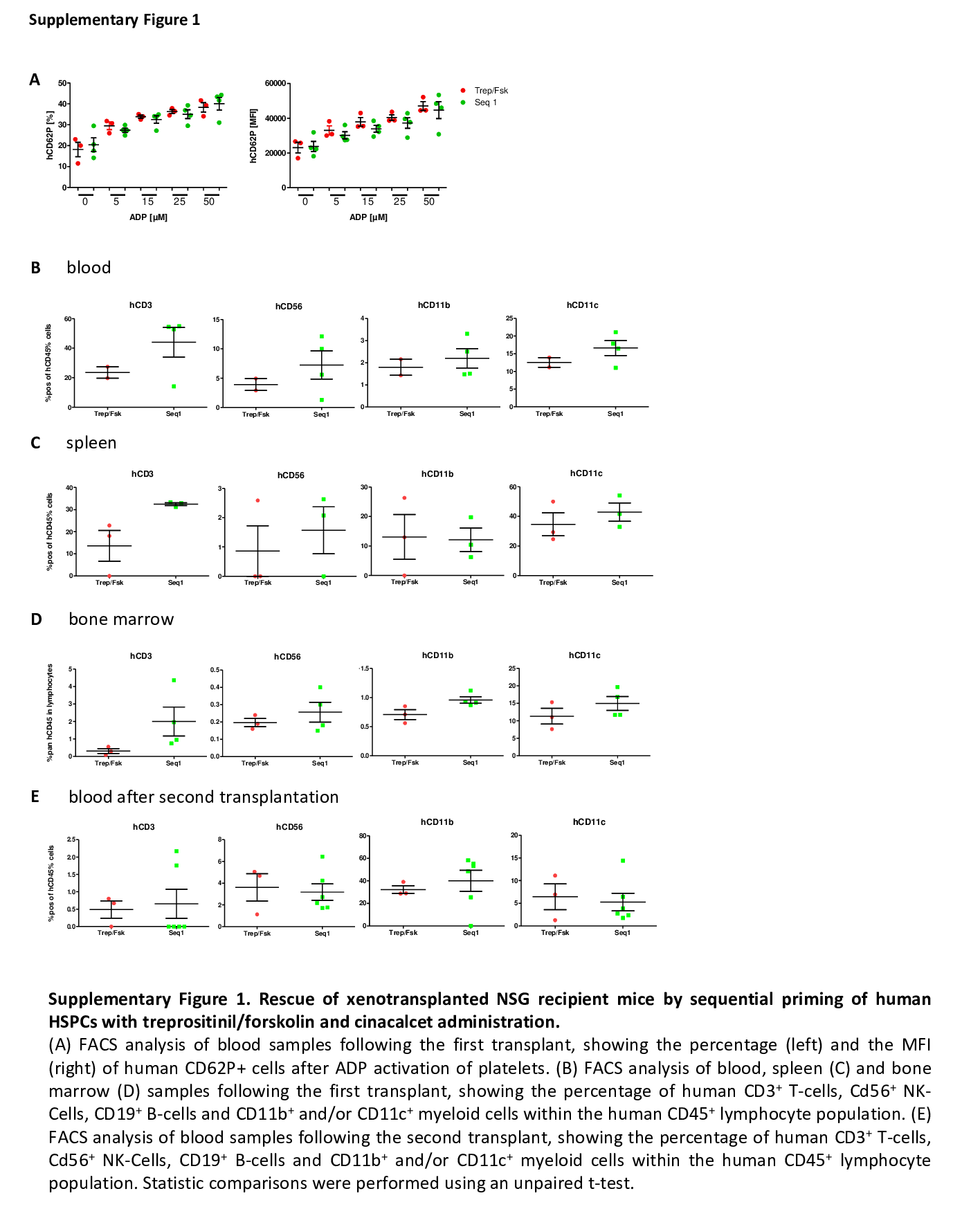

Supplement: Supplementary file 1 [file Image1.tiff]
